# Food hypersensitivity among asthmatic children – single center experience

**DOI:** 10.1186/2045-7022-3-S3-P37

**Published:** 2013-07-25

**Authors:** SS Al-Ani, S Al-Hammadi

**Affiliations:** 1 Pediatrics, Khorfakkan Hospital, Khorfakkan, United Arab Emirates; 2 Pediatrics, College of Medicine and Health Sciences, UAE University, Al-Ain, United Arab Emirates

## Background

The prevalence of asthma in children has increased in the last decades. One of the risk factors is sensitization to aeroallergens and food allergens, which contribute to severity of asthma and the problematic asthma that are not responding well to therapy. Children with food allergy noticed to have increased bronchial hyperreactivity (BHR) independent of respiratory symptoms.

Asthma is an uncommon manifestation of food allergy which is usually seen with other food-induced symptoms. Vapors or steam emitted from cooking food may induce asthmatic reactions.

Food-induced asthmatic symptoms should be suspected in patients with refractory asthma, history of atopic dermatitis, gastroesophageal reflux, food allergy or feeding problems as an infant, or history of positive skin tests or reactions to food.

## Methods

This studyincluded asthmatic children (n=659) attended pediatric asthma and allergy clinic at Khorfakkan hospital (1st Oct 2009 – 1st Mar 2011). Skin prick test (SPT) was done for 103 children with evidence of frequent exacerbation of asthma symptoms or refractory asthma. No antihistamine was given for at least 2 weeks before SPT.The SPT includes a series of inhaler (by Stallergens®) and food (fresh fruits or vegetables) allergens.

## Results

Out of 103 children, 63 (61.2%) are tested positive to at least one allergen. Forty-three (68.3%) are males. The highest rate of the positive tests are at the age group 5-12 years (27, 42.9%), followed by age group 2-5 years (19, 30.2%). This trend is the same between both sexes. Nineteen children are positive to 3 allergens (30.2%), 15 to 2 allergens (23.8%) and 11 to 1 allergen (17.5%). The commonest indoor aeroallergens are house dust mites (Der f 16%, Der p 13.5%) dog (11.5%), and cockroaches (10.9%). The commonest food allergens found are horse chestnut (20%), sesame (14%) and cow's milk (12%). Collectively, the indoor aeroallergens are twice positive as food allergens.

## Conclusion

Aeroallergens and food allergy are important causes of asthma exacerbations in children. This should always be considered in refractory asthma or asthma with frequent exacerbations.

## Disclosure of interest

None declared.

